# Notes and News

**Published:** 1906-04-14

**Authors:** 


### Notes and News.

The Swiss Government have notified the Powers concerned that they have arranged with the Russian Government that the conference for the revision of the Geneva Red Cross Convention of 1864 shall assemble at Geneva on the 11th of next June. The Conference had originally been fixed for August 1904, but the arrangements were cancelled owing to the outbreak of the Russo-Japanese war.

At the quarterly meeting of the Council of the Royal College of Surgeons, the Walker Prize for cancer research was awarded to Professor Jensen, of Copenhagen; and the Jacksonian Prize of 1905 to Reginald Cheyne Elmslie, M.S., F.R.C.S., for his essay on "The Pathology and Treatment of Deformities of the Long Bones due to Disease occurring during and after Adolescence."

A meeting was held at the residence of Lady Sudeley, Ham Common, in order that the cure of inebriety carried on at the Dadson Nursing Homes might be explained in connection with the movement for the establishment of a branch at Richmond. Archdeacon Sinclair, who said that the one great evil the clergy found that they had to contend with amongst all classes was the slavery of intoxication, declared that the aim in the Dadson Homes was to bring about not merely a mitigation, but an absolute eradication of the disease.

The Bishop of Southwark, writing on behalf of his colleagues, the acting chaplains of Guy's Hospital, and himself, urges upon the parochial clergy the great value of closer communication between themselves and hospital chaplains. Dr. Talbot points out that anybody who has had the least insight into the exacting and difficult duties of a hospital chaplain, obliged to handle with great rapidity a large number of changing and unknown cases, must be aware of the enormous help that is given to him, and therefore indirectly to the patient, if he starts with some knowledge, sympathetically and discreetly applied to him, of a particular case. He believes, moreover, that a very marked effect would sometimes be produced upon patients if they learnt that their clergy were in close touch with one another, had confidence in each other's ministrations, and cared enough about the patients to take pains in recommending and passing them on.

A conference on legislation in regard to children will be opened by the Lord Mayor at the Guildhall on Tuesday, May 22, and continued the following day. It has been convened by the Committee of the International Congress for the Welfare and Protection of Children, held in London in 1902, with a view to the discussion, in the light of their latest developments, of certain questions which were dealt with at the Congress. The following subjects will be considered in all their aspects, alike as to principles and details :—(1) The free feeding of school children; and (2) children's courts, probation officers, and remand homes. The Executive Committee of the Conference, of which Mr. W. G. Lewis, barrister-at-law, 8 Wells Street, Gray's Inn Road, W.C., is Secretary, includes Lady Frederick Brudenell Bruce, Sir William Chance, Bart., Dr. J. M. Rhodes, Miss Baker (of the Metropolitan Asylums Board), and Dr. Shuttleworth.

At the thirty-fifth Congress of the German Chirurgical Society, which took place in Berlin on April 4, mention was made of the severe loss the Society had sustained by the death of Professor Miculicz, of Breslau; and Professor Koch was elected an honorary member. The first day was devoted to the reports of the German surgeons who had been sent to the seat of war in Eastern Asia. The first paper, by Zoege Manteuffel, showed that the Russian Army Medical Corps was as little prepared for war as the army itself. The great distance from the seat of war, the hurry and scurry from the start, caused endless difficulties and muddle. The work was enormous. During every battle about one-third of the combatants were more or less seriously wounded; 2,700 medical men were employed in the Russian field hospitals and at the front. After an important battle they worked during the entire night, and did on an average 100 to 150 dressings each. The wounds inflicted by the small calibre arms of the Japanese allowed the adoption of conservative methods of treatment in most cases. Wound complications occurred mostly when the wounds were caused by shrapnels. Statistics of the wounded who were sufficiently recovered to return to the seat of war and take up active service again were given. These investigations reveal the fact that in some regiments 30 to 50 per cent. of the wounded were sent back to the front cured. Many surgeons read papers relating to war surgery, and on the whole the humane character of the modern forms of arms was very much questioned. Great interest was aroused by a patient shown who, after a fall on the back of the head, has been in a continuous comatose condition for one and a half years, while the normal functions of the body remain normal.

